# A new glance on root-to-shoot *in vivo* zinc transport and time-dependent physiological effects of ZnSO_4_ and ZnO nanoparticles on plants

**DOI:** 10.1038/s41598-019-46796-3

**Published:** 2019-07-18

**Authors:** Tatiana N. M. da Cruz, Susilaine M. Savassa, Gabriel S. Montanha, Juliane K. Ishida, Eduardo de Almeida, Siu M. Tsai, José Lavres Junior, Hudson W. Pereira de Carvalho

**Affiliations:** 1 University of São Paulo, Nuclear Instrumentation Laboratory, Center for Nuclear Energy in Agriculture, Piracicaba, 13416000 Brazil; 2 University of São Paulo, Cellular and Molecular Biology Laboratory, Center for Nuclear Energy in Agriculture, Piracicaba, 13416000 Brazil; 3 University of Sao Paulo, Center for Nuclear Energy in Agriculture, Plant Nutrition Laboratory, Piracicaba, 13416000 Brazil

**Keywords:** Permeation and transport, Plant physiology, Analytical chemistry, Transporters, Nanoparticles

## Abstract

Understanding nanoparticle root uptake and root-to-shoot transport might contribute to the use of nanotechnology in plant nutrition. This study performed time resolved experiments to probe Zn uptake, biotransformation and physiological effects on *Phaseolus vulgaris* (L.). Plants roots were exposed to ZnO nanoparticles (40 and 300 nm) dispersions and ZnSO_4(aq)_ (100 and 1000 mg Zn L^−1^) for 48 h. Near edge X-ray absorption spectroscopy showed that 40 nm ZnO was more easily dissolved by roots than 300 nm ZnO. It also showed that in the leaves Zn was found as a mixture Zn_3_(PO_4_)_2_ and Zn-histidine complex. X-ray fluorescence spectroscopy showed that root-to-shoot Zn-translocation presented a decreasing gradient of concentration and velocity, it seems radial Zn movement occurs simultaneously to the axial xylem transport. Below 100 mg Zn L^−1^, the lower stem tissue section served as a buffer preventing Zn from reaching the leaves. Conversely, it was not observed for 1000 mg Zn L^−1^ ZnSO_4(aq)_. Transcriptional analysis of genes encoding metal carriers indicated higher expression levels of tonoplast-localized transporters, suggesting that the mechanism trend to accumulate Zn in the lower tissues may be associated with an enhanced of Zn compartmentalization in vacuoles. The photosynthetic rate, transpiration, and water conductance were impaired by treatments.

## Introduction

The world population is estimated to reach 9.7 billion by 2050, raising serious concerns about global food, feed, fuel, and fiber demand. Food and feed are supplied by crops, while fuel and fiber come partially from oil. Thus, to ensure food security and offer a renewable source of platform molecules, farming must be more productive and efficient. Since most fertilizers are obtained from mining, and their sources are limited, their use must be as rational as possible. The plant nutrient demand is not constant along its life cycle^1^, it dramatically increases during certain phases like blooming and grain filling^2^. Therefore, fertilizers should meet the plant demand releasing nutrients according to its necessity. When regular fertilizers are broadcast in soils, most nutrients ionic species are adsorbed to soil colloidal particles, immobilized by microorganisms or leached. Only a fraction is used by the plants at the expense of plant energy^3^.

Zinc (Zn) is the least available micronutrient world wide^4^. Zn sulfate is the most common source of Zn^5–7^, specifically because of its higher solubility compared to oxides and carbonates, and lower cost contrasting to synthetic chelates and complexes^8^.

Understanding the mechanisms of Zn uptake and transport is a fundamental step that can subsidize the development of more efficient fertilizers. By uncovering the chemistry and physics behind these subjects, one might open the possibility of creating Zn sources that are easily taken up in the right moment with lower energy expenditure.

Zn plays important roles in plants metabolism such as the enzymatic activation^9^. It participates of growth regulation^10^, affecting root development, structure, and growth parameters. Zn transport is commonly described to happens predominantly through the symplast pathway by specific transporters^11,12^. This metal is also able to circulate in the apoplast space in the roots before reaching the Casparian strip^13^. Many genes are involved in the processes of Zn transport, as *ZIP*, *NRAMP*, *YSL*, *HMA*^13,14^. It seems that their expression varies according to the medium conditions^13,15–21^ and different plant tissues^14^.

In excess, typically higher than 400 mg kg^−1^ of Zn in dry mass of plant tissue, Zn is toxic to plants^22^. Zn toxicity has several implications in many metabolic processes. Since it is a constituent of special proteins related to DNA and RNA stabilization, in excess, it can cause genetically related disorders^23–25^. Before the appearance of toxicity symptoms, plants have some homeostatic defense mechanisms^13,26,27^. The altered Zn levels activate genes to avoid the excessive or the poor absorption and accumulation in plant tissues such as transcriptional factors, enzymes, channels, and transporters^28^.

Studies have shown that nanomaterials can promote seed germination^29,30^, boost plant defense system^31^ and enhance soil conditions^32^. Additionally, they can potentially be used for plant nutrition^33–37^. The ZnO nanoparticles (NP) enhanced the yield and growth of plants^35^, it increased the stem and root biomass in peanuts^38^, tomato^39^ and positively impacted the photosynthesis efficiency in *Arabidopsis*^40^. However, how NP releases Zn, its assimilation, transport, biotransformation and accumulation^37,41^ mechanisms in plant tissues are not fully understood. Currently, a knowledge bottleneck prevents the safe application of the NP-based fertilizers in crop production.

In this study, we combined X-ray spectroscopy and plant physiology to investigate how *Phaseolus vulgaris* (L.) model species (common beans) absorb, transport and store ZnO NPs and ZnSO_4_. The Zn transport was *in vivo* traced by X-ray fluorescence spectroscopy (XRF) and the storing mechanism was unraveled by X-ray absorption spectroscopy (XANES). Meanwhile, transpiration, CO_2_ photosynthetic rate and the transcriptional responses in several genes such as *ZIF1*, *MTP8*, *IRT3*, *HMA2*, *NRAMP3*, *NRAMP4*, and *MTP1* were monitored.

## Results and Discussion

### Root absorption and transport along the stem

Table 1 presents the fraction dissolved Zn from ZnO NPs and ZnSO_4(aq)_ in contact with roots and water control. The concentration of ZnO in the dispersion did not present a clear ef fect on the content of dissolved Zn. On the other hand, the amount of dissolved Zn increased in the presence of roots. This effect is likely caused by the action of organic acids exuded by roots. This effect is consistent with other authors who reported increased dissolution of NP under root exudates^42–44^. Experiments analyzing the bioavailability of copper ions from soil exposed to synthetic root exudates and nano Cu revealed an increase in the Cu^2+^ concentration in the soil solution^45^.

**Table 1:** Recovery of ZnSO_4_ and solubility of ZnO nanoparticles after 48 hours, and their standard deviation, under pristine aqueous dispersions and the same dispersions exposure to plant roots.

Treatments	Root Contact		Water	
	100	1000	100	1000
	(mg L^−1^)			
40 nm	30.1 ± 4.5^c^	20.7 ± 4.1^b^	9.6 ± 0.6^a^	8.4 ± 0.3^a^
300 nm	7.7 ± 0.9^a^	8.2 ± 1.1^a^	6.7 ± 0.4^a^	8.6 ± 0.5^a^
ZnSO_4_	106.1 ± 10.6^d^	1180.2 ± 11.7 ^f^	101.8 ± 1.5^d^	995.2 ± 23.3^e^

Values followed by the same letter do not present statistical difference according to Tukey Test at 95% probability.

Figure S1 shows a series of root pictures taken after 48 h root exposure to the 100 and 1000 mg L^−1^ nano ZnO dispersions, while Table S1 presents the characterization of the dispersed particle. In agreement to other authors^46^, we observed ZnO aggregates adsorbed on the root surface. The zeta potential (Table S1) remained negative, but its magnitude is reduced under the root influence. In addition to the sedimentation, this factor contributed to decrease the Zn concentration recovered in the liquid phase, as shown in Table S1. Thus, the actual Zn concentration in the rhizosphere, i.e. in the root nearby, is higher than the putative one. The aggregation and zeta potential are in line with the slight increase in average particle size determined by dynamic light scattering in Table S1.

**Figure 1:**
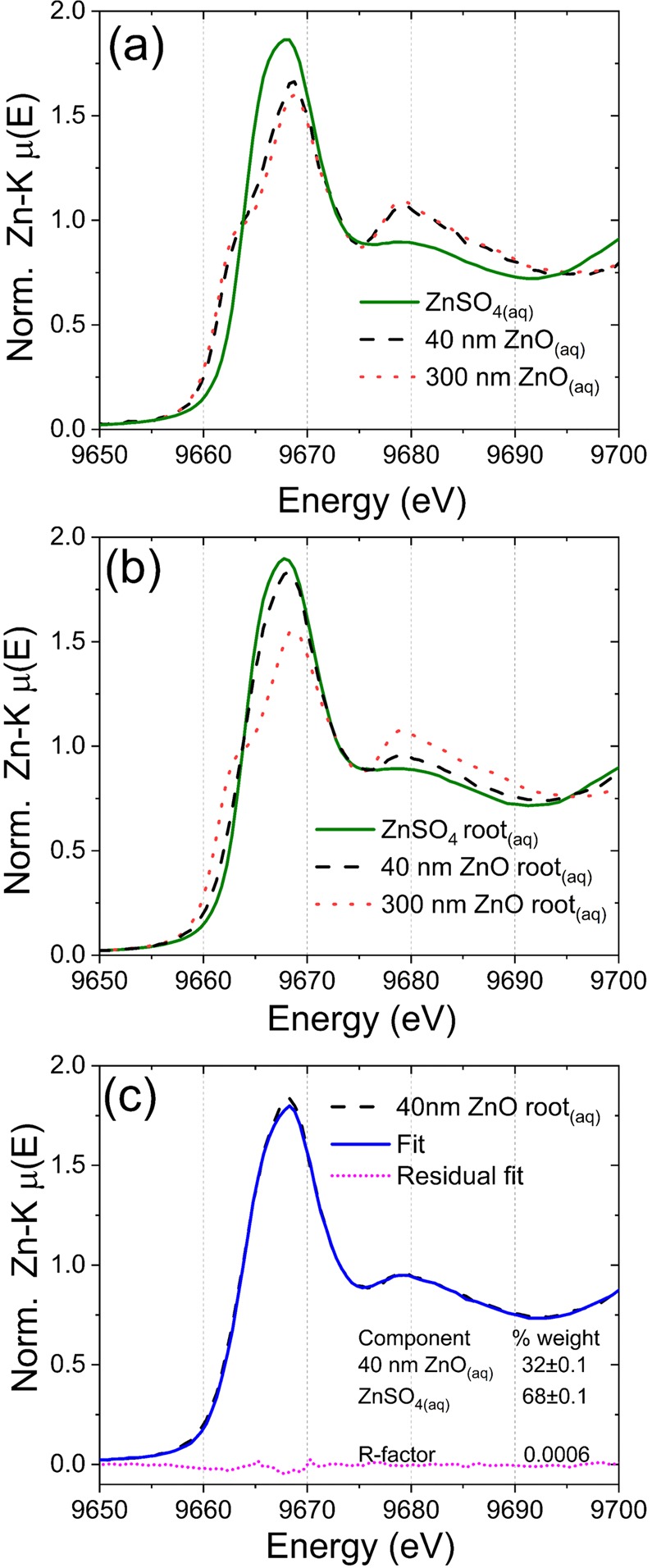
XANES spectra recorded for references and liquid aliquots collected from the 1000 mg L^−1^ solution and dispersions that remained 48 hours in contact with *P*. *vulgaris* roots; (**a**) spectra highlighting the different chemical environment for ZnSO_4(aq)_, 40 nm and 300 nm ZnO, (**b**) spectra for ZnSO_4_ solution and 40 nm and 300 nm ZnO dispersions that remained in contact with *P*. *vulgaris* roots, and (**c**) linear combination analysis for 40 nm ZnO uncovering that it was partially dissolved. The spectrum can be described as 68% ZnSO_4(aq)_ + 32% 40 nm ZnO with R-factors of 6 × 10^−4^.

XANES was employed to probe how roots influenced the chemical environment of dissolved and dispersed Zn. Figure 1(a) shows the XANES spectra recorded in an aliquot collected from 1000 mg L^−1^ ZnSO_4(aq)_, 40 nm and 300 nm ZnO solution, while Fig. 1(b) presents dispersions that were let in contact with roots for 48 hours. The different spectral features indicated that Zn chemical environment was not the same for all treatments. We did not detect any measurable change in the chemical neighborhood of ZnSO_4(aq)_ caused by roots.

Conversely, Figs 1(c) and S2(a) show that after 48 hours in contact with roots, the 40 nm ZnO was partially dissolved. The linear combination analysis of the XANES spectrum unravels that Zn could be described as a mixture of 68% soluble Zn and 32% 40 nm ZnO. The linear combination was carried out using Zn-malate coordination compound and ZnSO_4(aq)_. However, the spectrum of Zn-malate in solution was very close to the given by ZnSO_4(aq)_ (Supplementary Fig. S2(b)), the only difference remained in the feature at 9675 eV. Thus, we could not exactly assign the chemical nature of the soluble fraction. To address this issue, further studies employing high energy resolution XANES (HERFD-XANES)^47,48^ will be performed.

**Figure 2:**
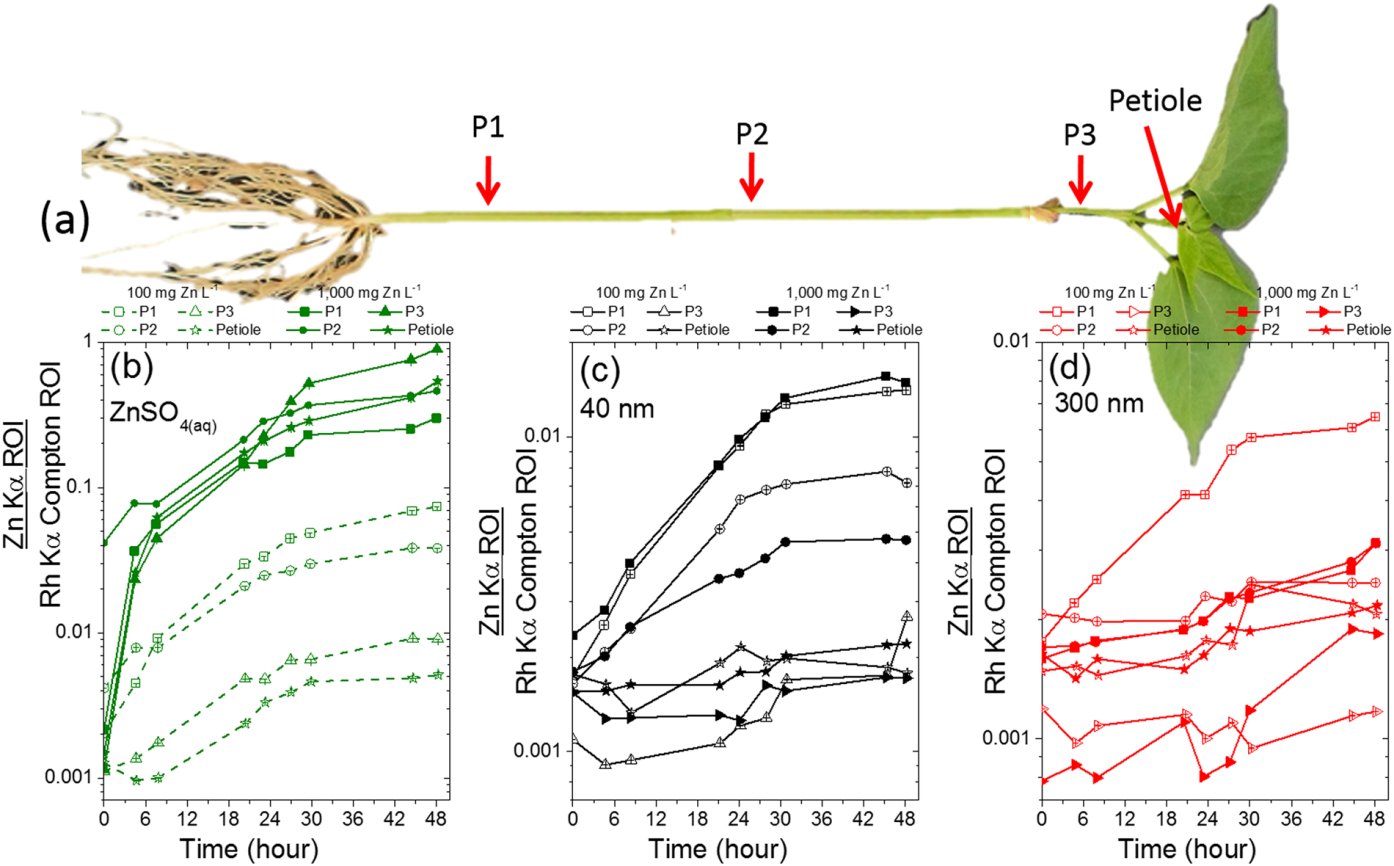
*In vivo* monitoring of the concentration of Zn in three points of the stem and in the petiole of *Phaseolus vulgaris* whose roots were immersed in 100 and 1000 mg Zn L^−1^; (**a**) the location in which the measurements were performed, and the uptake of Zn in plants exposed to (**b**) ZnSO_4(aq)_, (**c**) 40 nm ZnO, (**d**) 300 nm ZnO. The concentration of Zn decreases from root to shoot, except for 1000 mg Zn L^−1^ ZnSO_4(aq)_. The Zn counts at the time corresponding to zero hour were recorded immediately before the plants were exposed to the treatments.

The Zn content was monitored in three points of the stem (P1, P2 and P3) and in the petiole of the central leaflet as shown in Fig. 2(a). Figure 2(b–d) presents the Compton normalized counts of Zn in these four regions for *P*. *vulgaris* plants exposed to ZnSO_4(aq)_, 40 nm and 300 nm ZnO dispersions at 100 and 1000 mg Zn L^−1^. The Compton scattering normalization intended to correct thickness effects, making the detected X-ray fluorescence proportional to Zn concentration instead of capturing the total amount of Zn. This normalization step is important because the diameter of the stem can slightly varies from one plant to another.

One can observe that the Zn concentration decreased from P1 to the petiole, i.e. from root to shoot. The exception was the ZnSO_4(aq)_ at 1000 mg Zn L^−1^ that presented a different behavior, for example by the end of the experiment the concentration of Zn in the P3 point was higher than in P1, moreover the concentration of Zn in P2 was the same as in the petiole. This was confirmed in the biological repetition (Supplementary Fig. S3(a)) and by the quantification of Zn in shoot and root presented in Table S3.

Figure 2(b–d) also reveals that the concentration of Zn in the different points along the stem was less heterogeneous for 40 nm and 300 nm ZnO than for ZnSO_4_. The amplitude of the gradient of concentration from root to shoot decreased as follows ZnSO_4(aq)_ > 40 nm ZnO > 300 nm ZnO. This was also confirmed by the second biologic repetition shown in Supplementary Fig. S3(b,c).

Although the concentration of the dispersion in which the roots were immersed were 10-fold apart, it presented only a slight effect on the concentration of Zn found in the stem regions. The exception was the treatments with ZnSO_4(aq)_, after 48 h of exposure the number of Zn counts in the P1 of plants exposed to 1000 mg Zn L^−1^ solution was four-fold higher than that measured for the 100 mg Zn L^−1^ solution. In the case of the petiole, this figure was 100-fold higher for the 1000 mg Zn L^−1^ than for the 100 mg Zn L^−1^. These results corroborated the average Zn content determined at the root and shoot (Table S3).

**Figure 3:**
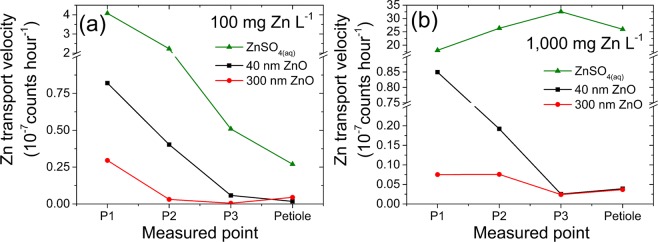
Zn uptake velocity in three points of the stem and petiole of *Phaseolus vulgaris* whose roots were exposed to (**a**) 100 mg Zn L^−1^ and (**b**) 1000 mg Zn L^−1^. The uptake velocity decreased from lower to the upper part of the stem, except for ZnSO_4(aq)_ at 1000 mg Zn L^−1^.

Figure 3 presents the uptake velocity along the shoot for plants exposed to (a) 100 mg Zn L^−1^ and (b) 1000 mg Zn L^−1^ treatment, the corresponding fitted slopes and correlation coefficients are presented in Table S2.

Regardless of the point of measurement and the concentration of the treatment, the Zn content followed a linear function of time. This finding is in agreement with a previous study involving more ZnO particles sizes and only a single point of the stem^37^. Figure S4 shows that the content of Zn in the stem also held a linear relationship with the concentration of soluble Zn in contact with roots.

In addition to the decreasing Zn content from root to shoot, the uptake velocity diminished. It also shows that the plant trended to store Zn in the lower tissues, as there is a gradient from P1 towards the petiole. Again, a divergent behavior was observed for ZnSO_4(aq)_ at 1000 mg L^−1^, for this treatment the uptake velocity increased in the upper tissues.

Figure S5 depicts XRF line scans showing the Zn content along the stem for the plants subjected to 1000 mg Zn L^−1^ treatments for 48 h. As shown in Fig. 2, the amount of Zn trended to decrease from root to shoot. In the plant treated with 1000 mg Zn L^−1^ ZnSO_4(aq)_ we observed that the Zn signal decreased then it increased again.

Under lower soluble Zn concentrations (ca. 7–100 mg Zn L^−1^, see Table 1), it seems that two mechanisms of Zn root to shoot transport coexist. An axial one, transporting the ions from the lower to the upper plant stem, this corresponds to the well-known xylem transport. However, the dilution effect, evidenced by the decreasing Zn concentration (Figs 2 and S5) and the decreasing velocity (Fig. 3), suggests the presence of a simultaneous radial movement. In this latter case, Zn would be pumped from the xylem towards the cortex tissues. These tissues would store part of the root absorbed Zn, either as defense strategy preventing it from reaching the leaves or as a nutritional pathway.

In principle, there are two hypotheses to explain the contrasting results found for the 1000 mg Zn L^−1^ ZnSO_4(aq)_ treatment. First, the high concentration of Zn readily available might have cause cell and tissue damage which lead to loss of function and the Zn ions could not be stored in the lower parts of the stem region. In a previous study on Mn toxicity, we observed that Mn excess induced tissue and intracellular disorganization. It increased vacuolation, elongated the chloroplast and the thylakoids were piled in a disorderly manner^49^. Second, the high amount of Zn quickly saturated the lower tissues of the stem which gradually stopped accumulating, whereas the upper tissues were still able to keep storing Zn.

Hence, the combination of *in vivo* X-ray probing the Zn concentration, Zn dissolution assays and chemical speciation of the dispersed/dissolved Zn show that the key factor controlling the uptake of Zn is rather the concentration of dissolved Zn than the concentration of the nanoparticle itself. The uptake of Zn from the 300 nm ZnO was smaller due to its lowest solubility. The amount of dissolved Zn, in turn, varied only slightly with the concentration of the dispersed ZnO and depended more on the nanoparticle size. Therefore, by defining the nanoparticle size, one can control the rate of Zn absorption.

### Zn in petiole and leaflet

**Figure 4:**
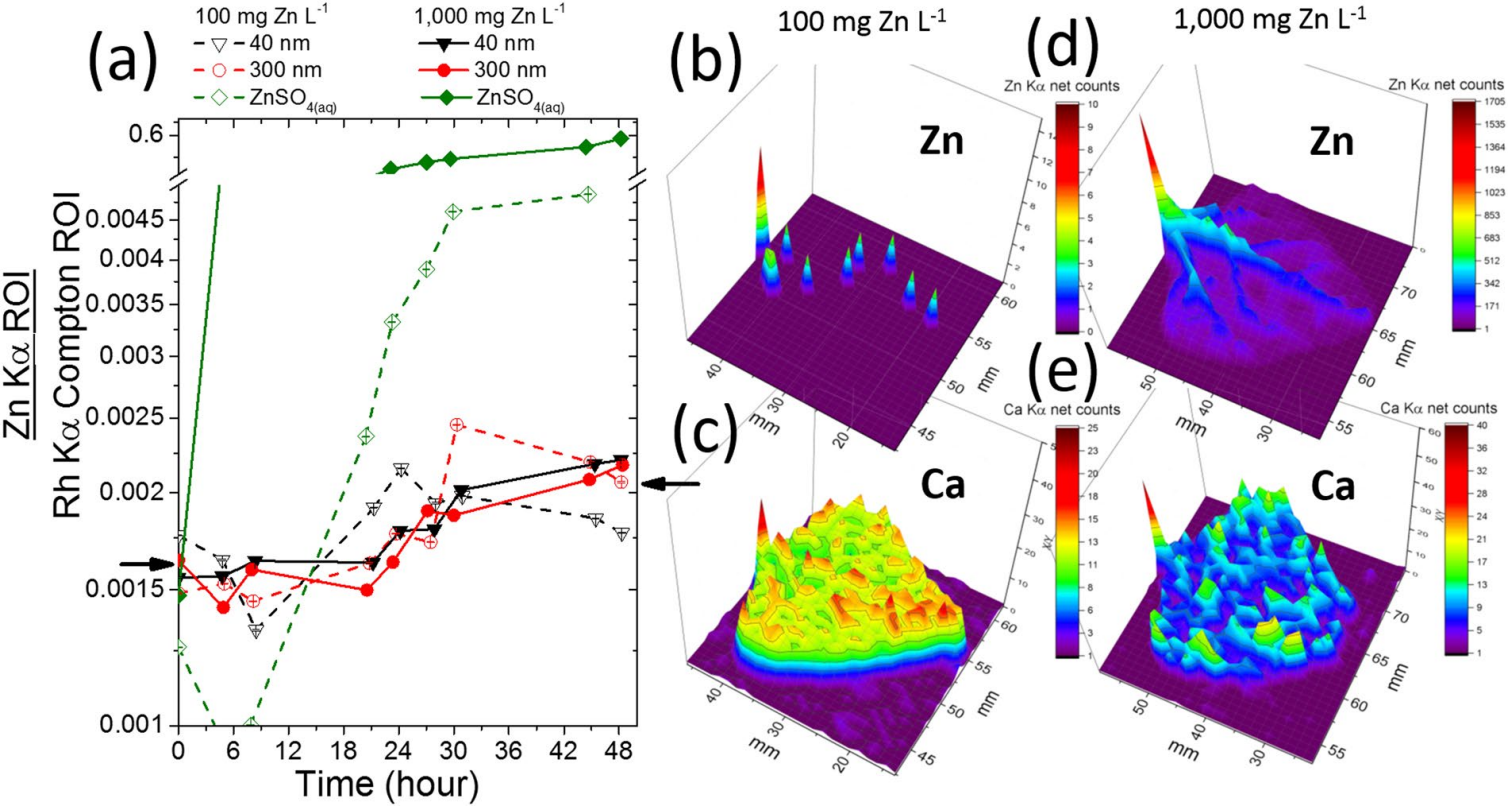
(**a**) Content of Zn in the petiole as function of time for plants exposed to 100 and 1000 mg of Zn L^−1^ of ZnSO_4(aq)_, 40 nm and 300 nm ZnO. The Zn counts at the time corresponding to zero hour were recorded immediately before the plants were exposed to the treatments; (**b**,**c**) spatial distribution of Zn and Ca, respectively, in the leaf whose plant was exposed to 100 mg Zn L^−1^ of ZnSO_4(aq)_, (**d**,**e**) spatial distribution of Zn and Ca, respectively, in the leaf whose plant was exposed to 1000 mg Zn L^−1^ of ZnSO_4(aq)_. The chosen leaflet was the central one in the first trefoil that was the petiole monitored by the XRF.

Figure 4(a) shows that the concentration of Zn in the petiole was more affected by ZnSO_4(aq)_ than ZnO NPs. As discussed above, the plant stem tissues might have acted as a buffer storing Zn and thus preventing it from moving upwards. Since the intensity of an X-ray fluorescence signal holds a linear relationship with the concentration of the analyte, one can state that the concentration in the petiole of the plant treated with ZnSO_4_ at 100 mg L^−1^ increased nearly 4-fold during the 48 hours of exposure. On the other hand, the concentration of Zn in the petiole of plants whose roots were immersed in 40 nm and 300 nm ZnO at 100 and 1000 mg L^−1^ increased by a factor less than 1.5 fold.

Figure 4(b–e) shows the spatial distribution of Zn and Ca in the central leaflet of the first trefoil (the one held by the XRF monitored petiole), (b) and (c) present maps for the plant treated with ZnSO_4_ at 100 mg L^−1^, while (d) and (e) for the plants treated with 1000 mg L^−1^. Since Ca is abundant and little mobile in the plant tissue, it was plotted here intending to assist the reader to visualize the leaf. Figure 4(b,d) show that Zn concentration decreases from the petiole to the leaf tip, this behavior was similar to that found for the stem. Zinc was mainly concentrated in the midrib of the leaflet treated with 1000 mg Zn L^−1^ (Fig. 4(d)), it can also be observed in the lateral veins.

The capacity of cotyledon leaves to behave as a Zn sink preventing the intoxication was evaluated, however the content of Zn found in the cotyledon leaves was smaller than that observed in the trifoliate leaves (not shown here). Thus, this hypothesis was rejected.

Aiming to investigate the chemical environment of Zn stored within leaves, *P*. *vulgaris* plants were treated with a lower concentration media (10 mg Zn L^−1^) for longer period (7 days). Table S3 shows that under these conditions the Zn content in the shoot increased (except the ZnSO_4(aq)_ at 1000 mg L^−1^) while it did not cause measurable injuries to the plants. This compromise enable to recorded good XANES signal and allowed accessing the Zn chemical species.

**Figure 5:**
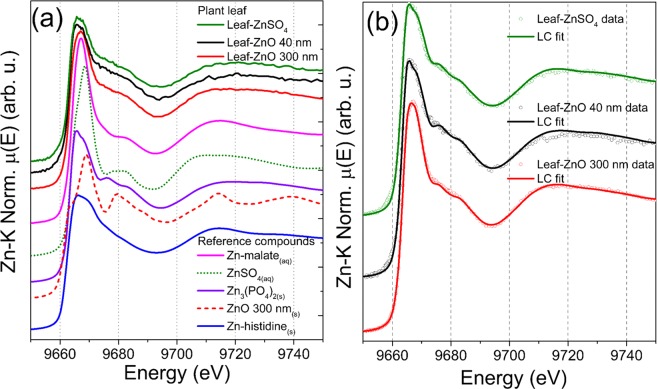
(**a**) Merged XANES spectra recorded in the leaves of *P*. *vulgaris* plants exposed to 10 mg Zn L^−1^ of ZnSO_4(aq)_, 40 nm ZnO and 300 nm ZnO for seven days, and reference compounds used in the linear combination analysis; (**b**) merged XANES spectra data and linear combination fitting. Regardless the source of Zn supplied to roots, Zn was mainly stored in a chemical form similar to Zn_3_(PO_4_)_2_ combined to minor fractions of Zn-histidine like compound.

Figure 5(a) shows the XANES spectra recorded at the leaves of the plants exposed to 10 mg Zn L^−1^ of ZnSO_4(aq)_, 40 nm and 300 nm ZnO, additionally this Figure displays the spectra of reference compounds used in the attempts of linear combination fitting (Fig. 5(b)). The spectral features were similar regardless of the treatment, except for a slightly higher white line feature for the 300 nm ZnO plant. The linear combination fitting (Table S4) indicated that the Zn present in the leaves could be described as a mixture. The major component was Zn phosphate (59–87%) and minor fractions Zn-histidine for ZnSO_4_, 40 and 300 nm ZnO. The spectrum for the 300 nm treated plant required the addition of minor fraction of Zn-malate reference compound. Zn phosphate, Zn-histidine^50,51^ and Zn-malate^37^ were previously reported as Zn chemical forms in plants. Although Fig. 4 and Table S3 showed that Zn reached the shoot, especially in the plants treated with 1000 mg Zn L^−1^ ZnSO_4(aq)_, the transporter expression discussed below was not correlated to the Zn content in these tissues.

### Effects of Zn treatments on plant physiology

**Figure 6:**
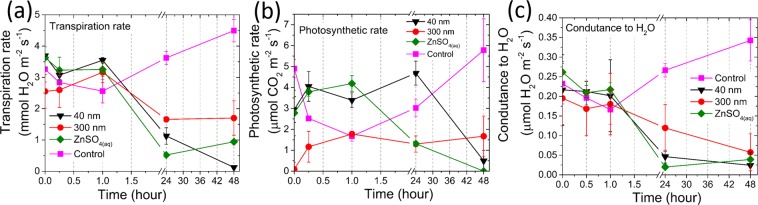
Physiological parameters obtained from IRGA measurements, (**a**) transpiration rate, (**b**) photosynthetic rate and (**c**) stomatal conductance, taken on the leaves in common bean plants exposed to 1000 mg of Zn L^−1^ from 40 nm and 300 nm ZnO dispersions, ZnSO_4(aq)_ solution and nutrient solution, for 48 hours.

The effects of Zn treatments on plant physiology were monitored using the infrared gas analyzer (IRGA). Figure 6 presents the measured (a) transpiration, (b) photosynthetic rates and (c) conductance to H_2_O as function of time for plants whose roots were immersed in 1000 mg L^−1^ ZnO (40 nm and 300 nm), ZnSO_4(aq)_, and a control plant that did not receive Zn.

Neither the transpiration rate nor the photosynthesis and conductance to H_2_O were affected during the first hour of treatment exposure. On the other hand, the measurement performed 24 hours later showed that the transpiration rate decreased whereas the photosynthetic rate was only reduced in the plants treated with ZnSO_4(aq)_. Finally, past 48 hours of exposure, the photosynthesis of the plants treated with 40 nm ZnO also decreased. At the end of the experiment, the leaves of plants treated with 1000 mg L^−1^ ZnSO_4(aq)_ became wilted.

In agreement with the results shown in Figs 2–4, the IRGA data indicated that Zn coming from ZnO nanoparticles source takes more time to be translocated by plants in exceeding amount. According to Supplementary Fig. S4, this might be related to the slow release of Zn ions from the ZnO nanoparticles compared to the fully available ZnSO_4_.

Figure S6(a,b) present the transpiration rate as function of the content of Zn in P1, P2 and P3 positions of the stem. The attempts of establishing a linear fit of the transpiration and the Zn content resulted in a R^2^ coefficient of determination ≤0.60. The plot for the nanoparticles treatments (Fig. S6(b)), i.e. low available Zn in the dispersion, suggested that the Zn content in the upper part of stem affects the transpiration rate more than the Zn the lower stem.

The data in Figs 4(a), 6(a) and S6(c) suggest negative correlation between the Zn content in the petiole and the transpiration rate on leaves. The damages in the upper structure can be resulted from cell plasmolysis and disruption^52^ which affects the photosynthetic rate and conductance to H_2_O (Fig. 6(b,c), respectively). Zinc toxicity might hamper CO_2_ assimilation rate and stomatal conductance^53^. However, it is still poorly understood whether photosynthesis inhibition as well as an impaired water-plant status, and thus stomatal limitations, are one of the main causes of heavy-metal toxicity (including Zn^2+^) in the shoots and roots^54,55^. Some evidence suggests that plant exposure to high metal concentrations in the substrate reduces physiological parameters such as plant–water relationships, and, in particular, heavy-metal toxicity has been stated to reduce stem and root hydraulic conductivity, and decrease xylem and leaf-specific hydraulic conductivity^53,54,56^.

For 300 nm ZnO, the lower concentration in the petiole (Fig. 2 (d)) can explain why the deleterious effects promoted by this treatment were less steep than those observed for ZnSO_4_ and 40 nm ZnO.

Thanks to the control experiments, one can hold that the increasing concentration of Zn in the root and shoot was responsible for slowing down the plant metabolism, corroborating some authors that found reduction on photosynthetic rate and stomatal conductance in response to Zn^+^2 excess^53^. The transpiration rate at the leaves decreased before the Zn concentration in the petiole started increasing, it means that the transpiration might primarily respond to the increase of Zn content in the bottom parts of plants.

Conversely, the photosynthesis was impaired by the increasing Zn content in the leaflet. A surprising piece of information regards the amount of Zn necessary to trigger the deleterious effects on photosynthesis. The photosynthetic rate in the plants treated with ZnO was diminished by an increase of ca. 50% of the Zn concentration.

**Figure 7:**
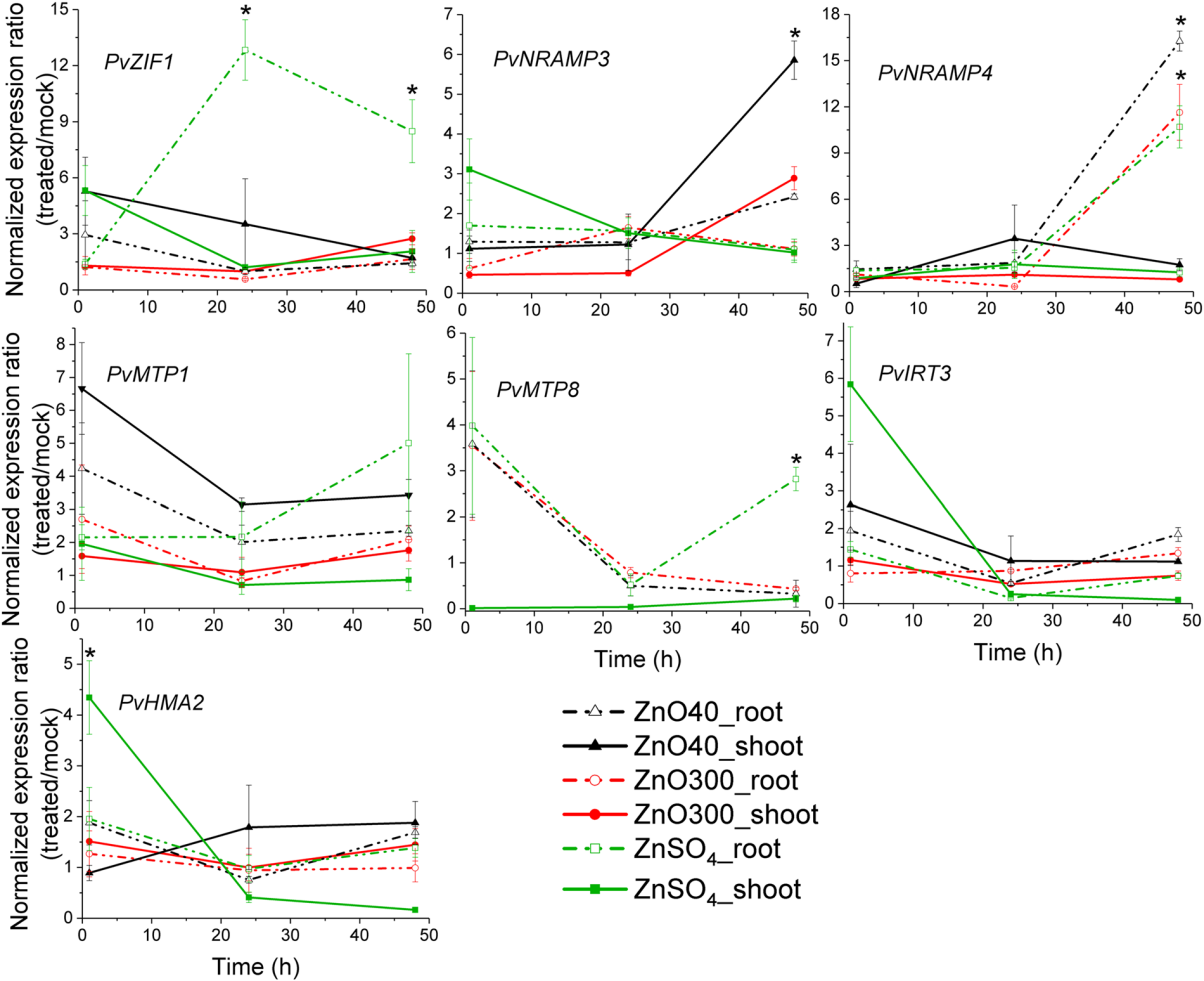
Effect of ZnSO_4(aq)_ and ZnO NPs on expression profiles of metal transporters. Relative transcriptional levels of *PvZIF1*, *PvNRAMP3* and *-4*, *PvMTP1*, *-8*, *PvIRT3* and *PvHMA2*, in shoots and roots of *P*. *vulgaris* seedlings submitted to 1000 mg/L ZnSO_4(aq)_, 40 nm and 300 nm ZnO NP for 1, 24 and 48 h. Values correspond to the averaged expression data shown as fold change between treated and control plants across three biological repetitions containing two seedlings each. Error bars denote standard errors (SE). *Indicates significant differences compared to the mock treatment by Student’s t-test with equal variances.

Carriers act on maintaining the balance of metals within the cells. Therefore, our efforts focused on understanding whether NPs would be able to alter the transcriptional regulation of genes encoding transporters involved in Zn homeostasis. We quantified the gene expression of some members of transporter families in *P*. *vulgaris* while the plant roots were immersed in ZnO NPs dispersions and ZnSO_4(aq)_. These analyses included the homolog genes in *P*. *vulgaris* genome encoding to the zinc-regulated transporters IRT3, HMA2 (Heavy Metal ATPase 2), ZIF1 (Zinc-Induced Facilitator1), MTP1 and 8 (Metal Tolerance Proteins 1 and 8) and NRAMP3 and 4 (Natural Resistance-Associated Macrophage Protein 3 and 4). The results are presented in Fig. 7. *PvIRT3* and *PvHMA2* showed similar expression patterns, being negatively regulated in shoots after 1 h of ZnSO_4(aq)_ exposure. The expression of the metal carriers *PvZIF1*, *PvMTP1* and *PvMTP8* were more pronounced in roots than in shoots, with a peak after 24 h of treatment with ionic Zn. *PvNRAMP4* expression was similarly modulated by ZnSO_4(aq)_ and NPs, showing a positive regulation in aerial part within 48 h of root exposure. The transcriptional levels of the vacuole-localized transporter *PvNRAMP3* significantly enhanced in shoots after 48 h of 40 nm ZnO treatment.

The balance of Zn concentration in the cells is maintained by a complex and tightly regulated action of members from Zn transporters families. Plants exposed to ZnO NPs presented changes in the transcriptional levels of HMA2 which is largely localized in the vasculature to promote efflux of Zn into xylem^13,28^. The carriers NRAMP3, −4, MTP1 and −8 are localized at the tonoplast and act on the translocation of essential metals through vacuole^57^. The sequestration of Zn into the vacuoles seems to be a strategy developed by the plant to prevent Zn reaching at the most metabolic active regions and thus delayed the intoxication^58,59^.

Post-Zn exposure caused a decrease in PvHMA2 expressions in the shoot, accompanied by heightening of the transcriptional response of translocators located at the tonoplast (NRAMP3, −4, *PvMTP1 and −8*) (Fig. 7). The data suggested a depletion of free Zn in the cytosol in favor of the storage of this metal in vacuoles, and a potential reduction of the efflux into the stem xylem. It could avoid the excessive import of Zn into the vasculature^60^. Such observation further supports the decrease of Zn concentration and the uptake velocity from the lower to the upper part of the plant (Figs 2 and 3). In addition, we observed that PvNRAMP3 showed sensitivity to 40 nm ZnO NPs in roots, no change occurred in shoots or upon the treatment with 300 nm ZnO NP (Fig. 7). This discrepancy may be related to the higher solubility of 40 nm ZnO nanoparticles in presence of roots (Table 1). This is possibly driven by organic exudates released into the rhizosphere by the plants.

In summary, our experiments showed that the solubility of NPs dispersion did not present great changes with the increase of the concentration. Conversely, it was more dependent on particle size. The 40 nm ZnO was more soluble than 300 nm in presence of roots. This is a key parameter governing the uptake rate. Literature shows that the ZnO solubility does not follow a linear function of the particle size. Thus, by matching the particle size together with the plant cycle length, one might be able to control the rate of Zn release according to the plant demand. The next step to accomplish such task involves Zn release experiments in soil conditions.

The XRF traced kinetics and the line scans obtained after the uptake experiments showed that the plant trended to accumulate Zn in the lower tissues. This suggests a type of buffer mechanism that retards Zn reaching the shoot and by consequence the photosynthetic machinery. Nevertheless, the combination of XRF and IRGA showed that a sudden increase of nearly 50% in the Zn content in the petiole, thus an even smaller increment in the leaflet cells, was already enough to decrease the photosynthetic rate. Regardless the source of Zn supplied through roots, in leaves, zinc was found as a mixture whose major component was identified as Zn_3_(PO_4_)_2_.

The results indicate that differences in gene expressions and leaf gas exchange modulated by Zn supply are related to Zn translocation or distribution of root-applied Zn along the plant tissues. Differential plant responses to Zn supply are a possible consequence of variation in nutrient uptake efficiency and long distance transport to shoots, which is modulated not only by the source, but also by the Zn availability.

## Methods

### Nanomaterials

The 40 nm NP was purchased as powder from M K Impex Corp (Canada), and the 300 nm NP was obtained as aqueous dispersion with surfactants from Agrichem Company (Brazil). These materials were used to prepare aqueous dispersions of 100 and 1000 mg L^−1^ of Zn in distilled water. The powder NP was sonicated with a Fisher Scientific Model 750 Sonic Dismembrator, operating at 60 W for three cycles of one minute, with 30 seconds of interval between each cycle.

### Evaluation of ZnO dissolution

To evaluate the solubility of nanomaterials, 50 mL of 1000 mg L^−1^ were prepared and the 40 nm NPs were sonicated as described above. Then 1 mL was transferred to vials and centrifuged at 14,007 g for 60 minutes. Right after, 15 μL of the supernatant was pipetted twice on the top of X-ray polypropylene film. The Zn concentration was determined by energy dispersive XRF (Shimadzu, EDX-720). For all measurements mentioned herein using this equipment, the Rh X-ray tube operated at 50 kV and auto-adjusted current to a maximum of 30% detector dead time. The acquisition time was 200 s. For quantification we used a set of external standards for calibration under thin condition (namely, the Zn X-ray fluorescence intensity is directly related to this element concentration by its sensitivity). The Zn intensities of standards and samples were corrected by the Ga internal standard at 50 mg L^−1^.

### *In vivo* Zn content monitoring

*Phaseolus vulgaris* seeds (variety BRS Estilo) were sown in vermiculite and watered daily with 20% strength modified Hoagland solution. The plants were transplanted to sample holders with 100 and 1000 mg of Zn L^−1^ of nano ZnO dispersions or ZnSO_4(aq)_ solution when they presented the first trefoil fully expanded. They were kept in a growth room, at 27 °C and 12 hours photoperiod under LED lamps illumination, supplying 250 μmol photons m^−2^ s^−1^. For the XRF measurements, the samples were loaded into equipment and then returned to the growth room. Control plants were kept in deionized water.

The Zn uptake was traced using an Orbis PC EDAX (USA) X-ray fluorescence spectrometer, where the x-rays were provided by a Rh anode operating at 45 kV and 900 μA. The x-ray beam had 1 mm in diameter, and it was collimated by a set of slits. It was used a primary 25 μm Ni filter to improve the signal to noise ratio. The XRF photons were detected by a 50 mm^2^ SDD detector, and the dwell time was 120 s, with a dead time smaller than 3%. The distance between the sample and the source was 10 mm, and no signs of sample burning were observed as function of time. The measurements were done using two biological replicates. The setup used in these measurements is shown in Supplementary Fig. S7. The XRF measurement uncertainty was calculated as described in the Supplementary Information.

### Effects of roots on the stability of suspended particles

The nanoparticles were dispersed in distilled water (100 and 1000 mg L^−1^ of Zn) by sonication as described above. To emulate the conditions described for the kinetic experiment, 50 mL of the dispersions were transferred to plastic pots. The plants were transferred to these pots and the roots remained in contact with dispersions for 48 h. Another group of pots containing the dispersions without plants was kept as controls.

The pH was measured using a Tec-2 pH meter (Tecnal, BR). After 48 h, aliquots of the supernatants were removed for X-ray diffraction, X-ray fluorescence analysis, Dynamic light scattering and Zeta potential. The details of each analysis are described below.

X-ray diffraction patterns were recorded using a PW 1877 diffractometer (Philips, The Netherlands) using Cu Kα radiation. The suspended aliquot of 0.1 mL was successively dripped on the sample holder glass surface. Multiple drippings, intercalated by drying, were performed in order to gather enough material, the total volume added ranged from 1.9 to 4.6 mL depending upon suspended concentration in the samples. The crystallite size was determined by the Scherrer equation using the peak full width at half maximum after the subtraction of the instrumental broadening. The value of the Scherrer constant was 0.94.

To record dynamic light scattering and zeta potential, we used a ZetaSizer ZS90 analyzer (Malvern Instruments, UK). The measurements were performed in triplicate, for each biological replicate, at room temperature. The sample dilutions in deionized water are presented in Table S1.

The concentration of Zn in suspension was determined by XRF (Shimadzu, EDX-720). A set of six Zn working solutions from 0 to 953 mg L^−1^ were used for external calibration. Fifteen microliters of standards and samples were pipetted on an X-ray cup external side (3577 micro X-cell, Spex Ind. Inc.) covered with 6 micrometers polypropylene film (P/N: FPP25-R3, VHG Labs, USA), and dried at 60 °C in a laboratory oven. Ga was used as internal standard at 50 mg L^−1^.

### Zn content along the plant stems and concentration of Zn in plant tissues

After the *in vivo* XRF measurements, the roots and leaves were detached using a metallic scalpel. The stems were frozen in liquid N_2_, placed on the surface of XRF sample holder covered with polypropylene. To prevent them from moving during the scan, the samples were stuck with polyamide scotch tape. The samples were loaded in the Orbis PC EDAX (USA) X-ray fluorescence spectrometer. The Zn content along the stem (line scan) was determined using a 1 mm wide X-ray beam delimited by slits. The 25 μm Ni primary filter was employed to improve the signal to noise ratio. The anode operated at 45 kV and 500 μA, 32 XRF spectra were recorded per line, the dwell time was 20 s. The XRF photons were detected by a 50 mm^2^ SDD detector.

For the quantitative analysis, a set of six Zn standards were prepared by adding known amount of Zn (1001 mg L^−1^ standard solution, AAZN1000V, Specsol, Brazil) in cellulose binder (3642, Spex, USA). After drying at 60 °C in a laboratory oven, the standards were fully homogenized in an agata mortar. The concentration of Zn in the standards range from 0 to 1800 mg kg^−1^. The common beam stems and roots were dried at 105 °C for 12 h and homogenized using the cryogenic grinding.

One hundred milligrams of the standards and samples were transferred to an X-ray cup (3577 micro X-cell, Spex, USA) sealed with 6 micrometer polypropylene film (VHG Labs). The samples were manually pressed using a glass rod. The samples were analyzed using an X-ray fluorescence EDX 720 Shimadzu spectrometer.

### Zn spatial distribution in leaves

After the *in vivo* experiment, the leaves from plants exposed to ZnSO_4(aq)_ with 100 and 1000 mg L^−1^ were dried at 60 °C in an oven for 72 hours. The leaves (Supplementary Fig. S8) were placed on the top of a polyamide thin film and assembled in a sample holder. Maps were recorded using a 1 mm X-ray beam delimited by a pinhole. A matrix of 64 × 50 pixels was employed in the mapping. X-rays were generated by a Rh anode operating at 40 kV and 300 μA under vacuum atmosphere. The distance between the sample and the X-ray source was 10 mm. The XRF photons were detected by a 50 mm^2^ SDD detector, the dwell time was 1 s per point, the dead time was smaller than 3%.

### Zn chemical speciation

The Zn-K edge X-ray absorption spectroscopy (XANES) measurements were carried out at the XAFS2 beamline, at LNLS (Campinas, Brazil). In this station, synchrotron radiation is produced by a bending magnet dipole, then a cylindrical Rh coated mirror rejects the higher harmonics and vertically collimates the beam. The suitable energy for Zn K edge was selected by a Si (111) double crystal monochromator. And a second Rh coated mirror focalizes the X-rays to a spot nearly 500 μm on the sample position.

The plants were cultivated in 10 mg Zn L^−1^ dispersions and solution for seven days. The 40 nm and 300 nm ZnO were supplied by Nanophase (USA) and Agrichem (Brazil), respectively. The XANES measurements were using detached leaves. The leaves were previously shock frozen using supercooled isopentane. The measurements were performed in fluorescence mode using a Canberra 15 element Ge solid state detector. Each near edge X-ray absorption spectra (XANES) was acquired in nearly 20 minutes and three spectra per point per sample was measured. To help preventing radiation damage a cryojet was also used, the setup at XAFS2 is shown in Supplementary Fig. S9. The photon flux density on the sample was 2.78 × 10^9^ photons s^−1^ mm^−2^ at 7 keV.

The spectra were energy calibrated and normalized using Athena program within the IFEFFIT package. The linear combination analysis was performed on the normalized data using the Athena program from -20 up to 70 eV relatively to the E_0_. The fit disagreement was expressed in terms of the R-factor as described in the Supplementary Information.

Visual symptoms of radiation damage (scorching) were not observed. However, for one the samples we noticed a <6% reduction on the white line intensity for the third spectrum of the series, which could be an indicative of radiation damage. Therefore, the linear combination analysis was performed using both three merged spectra and the first spectrum of the series (Table S4), regardless the strategy the percent composition fall within the error bars variation. Additionally, the individual non-normalized scan spectra are shown in Fig. S10.

For the XANES measurements in the solution and dispersion that remained in contact with the roots, we sampled aliquots and loaded them in the sample holders shown in Supplementary Fig. S11. Additionally to the samples, we recorded XAS spectra for Zn-malate, pristine nano ZnO and commercial ZnSO_4_.

### Infrared gas analyzer experiment

Physiological evaluations of gas exchanges were performed in the middle leaflet of the first expanded trefoil, 5 times, with no exposure to treatments, after 15 minutes, 60 minutes, 24 hours and 48 hours of exposure. For this purpose, evaluations of gas exchange consisted of non-destructive analyses using a portable gas exchange device (Infra Red Gas Analyzer – IRGA, Li-6400XT, LI-COR Inc.). The following were determined: CO_2_ assimilation rate expressed by area (A - μmol CO_2_ m^−2^ s^−1^), transpiration (E - mmol H_2_O m^−2^ s^−1^) and stomatal conductance (gs - mol H_2_O m^−2^ s^−1^). The initial conditions imposed for measurements were 1000 μmol m^−2^ s^−1^ of photosynthetically active radiation (PAR), provided by LED lamps, air CO_2_ concentration of 400 ± 20 μmol mol^−1^, and a chamber temperature of 25 °C, according to other studies^61,62^. The control plants were exposed to deionized water for 48 h and subjected to the same measurements.

### Quantitative RT-qPCR analysis

Treatments with 1000 mg of Zn L^−1^ of 40 nm, 300 nm ZnO dispersions and ZnSO_4_ solution were carried out using *P*. *vulgaris* seedlings whose first trifoliate leaf was fully expanded. The seedlings were cultivated in vermiculite and previously irrigated with deionized water. Then, the roots of seedlings were immersed in the treatments for 1, 24 and 48 h. For the control, the plants were immersed to deionized water and submitted to the same procedures as those treated. Total RNA was extracted using a CTAB/LiCl based-protocol as previously reported^63^. Genomic DNA was removed from the RNA samples by treatment with DNase I, Amplification Grade (Sigma-Aldrich. Cat No. AMPD1-1KT). RNA was quantified with a NanoDrop ND-1000 spectrophotometer (NanoDrop Technologies). For RT-qPCR analysis, a primer pair designed for the *P*. *vulgaris* gene encoding to actin protein was used for the internal control^64^. The first-strand cDNA was synthesized with a High Capacity cDNA Reverse Transcription Kit (Applied Biosystems, Code 4368814), and qPCR was performed with a SyBR® Green PCR Master Mix kit (Applied Biosystems, Code 439155). Each reaction was run in a 10 µL volume which contained 2 µL cDNA (~20 ng), 5 µL of power SYBR mix, 0.2 µL each primer to a final concentration of 250 nM. PCR cycling was 95 °C for 15 min, followed by 40 cycles of 10 s at 95 °C, 1 min at 60 °C. All the gene expression was calculated with the standard curve method, established from a series of six dilutions using samples with the highest expression level for each gene. The expression data for each gene was normalized by the internal reference control (actin)^64^. The expression values are shown as averaged between treated/control plants. The primers specifically designed for common bean transporter genes are listed in Table S5. All experiments were repeated at least three times with at least two technical replications each. For statistical analysis, the t-test was performed across the replicates.

## Supplementary information

A new glance on root-to-shoot in vivo zinc transport and time-dependent physiological effects of ZnSO4 and ZnO nanoparticles on plants
