# Supplementary material for: A new glance on root-to-shoot in vivo zinc transport and time-dependent physiological effects of ZnSO4 and ZnO nanoparticles on plants
Source: Sci Rep. 2019 Jul 18;9:10416. doi: 10.1038/s41598-019-46796-3 (PMC6639404; doi:10.1038/s41598-019-46796-3)
Supplement: Supplementary file 1 — A new glance on root-to-shoot in vivo zinc transport and time-dependent physiological effects of ZnSO4 and ZnO nanoparticles on plants [file 41598_2019_46796_MOESM1_ESM.pdf]

Supplementary Information

A new glance on root-to-shoot *in vivo* zinc transport and time-dependent physiological effects of $ZnSO_4$ and ZnO nanoparticles on plants

Tatiana N. M. da Cruz[1], Susilaine M. Savassa[1], Gabriel S. Montanha[1], Juliane K. Ishida[2], Eduardo de Almeida[1], Siu M. Tsai[2], José Lavres Junior[3], Hudson W. P. de Carvalho[1*]

[1]University of São Paulo, Nuclear Instrumentation Laboratory, Center for Nuclear Energy in Agriculture, Piracicaba, 13416000, Brazil
[2]University of São Paulo, Cellular and Molecular Biology Laboratory, Center for Nuclear Energy in Agriculture, Piracicaba, 13416000, Brazil
[3]University of Sao Paulo, Center for Nuclear Energy in Agriculture, Plant Nutrition Laboratory, Piracicaba, 13416000, Brazil

Contents

Figure S1 2
Figure S2 3
Figure S3 3
Figure S4 4
Figure S5 4
Figure S6 5
Figure S7 6
Figure S8 6
Figure S9 7
Figure S10. 8
Figure S11. 8
Table S1. 9
Table S2. 10
Table S3. 11
Table S4. 12
Table S5. 13
Uncertainty of XRF measurements 14

Optical images

The plants were exposed to ZnO dispersions using the same procedure described in the section "*In vivo* Zn concentration monitoring" of the main manuscript. After 48 h they were removed and the roots were randomly collected for analysis. While they were still fresh, the photographs were recorded using a Hirox 3D microscope model KH 8700.

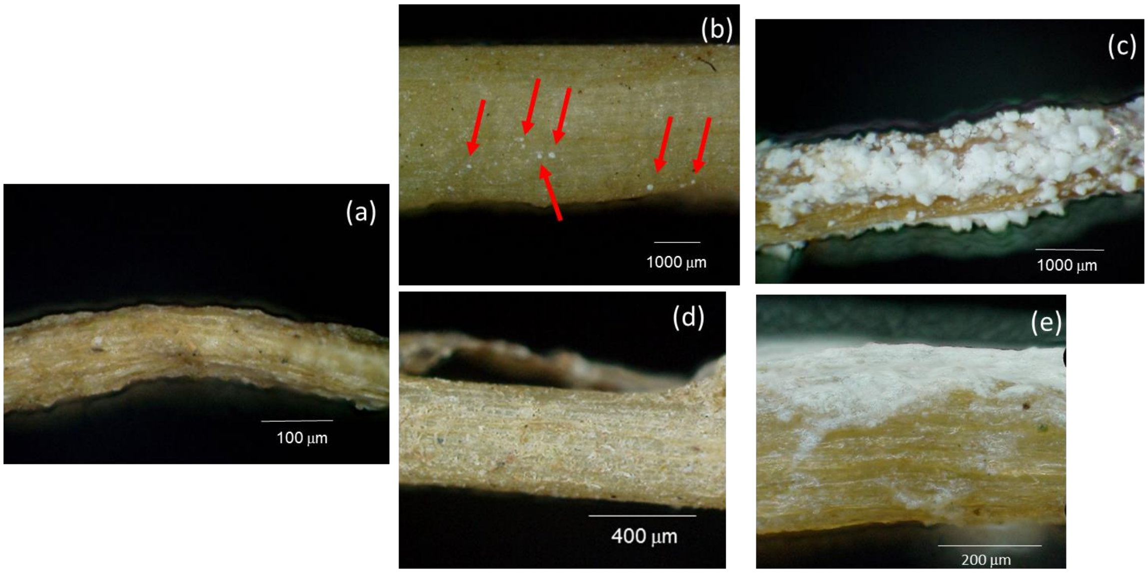

Figure S1. Optical images of *P. vulgaris* roots exposed to (a) deionized water, (b) 100 mg L^{-1} of 40 nm ZnO, (c) 1000 mg L^{-1} of 40 nm ZnO, (d) 100 mg L^{-1} of 300 nm ZnO and (b) 1000 mg L^{-1} of 300 nm ZnO, The roots remained in contact with water and ZnO dispersions for 48 h. The red arrows show small ZnO aggregates.

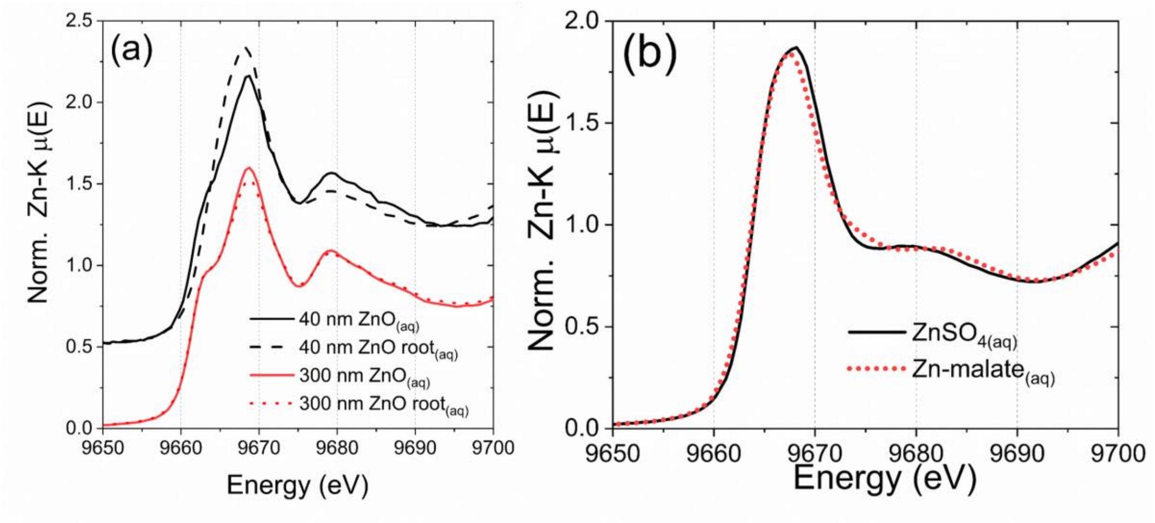

Figure S2. (a) Zn-K XANES spectra for pristine 40 nm and 300 nm ZnO aqueous dispersions and aqueous dispersions that remained in contact with roots for 48 h. The samples were measured in XRF geometry without dilution at 1000 mg Zn L^{-1}. (b) XANES spectra for aqueous $ZnSO_4$ and Zn-malate reference compounds recorded at 1000 mg Zn L^{-1}.

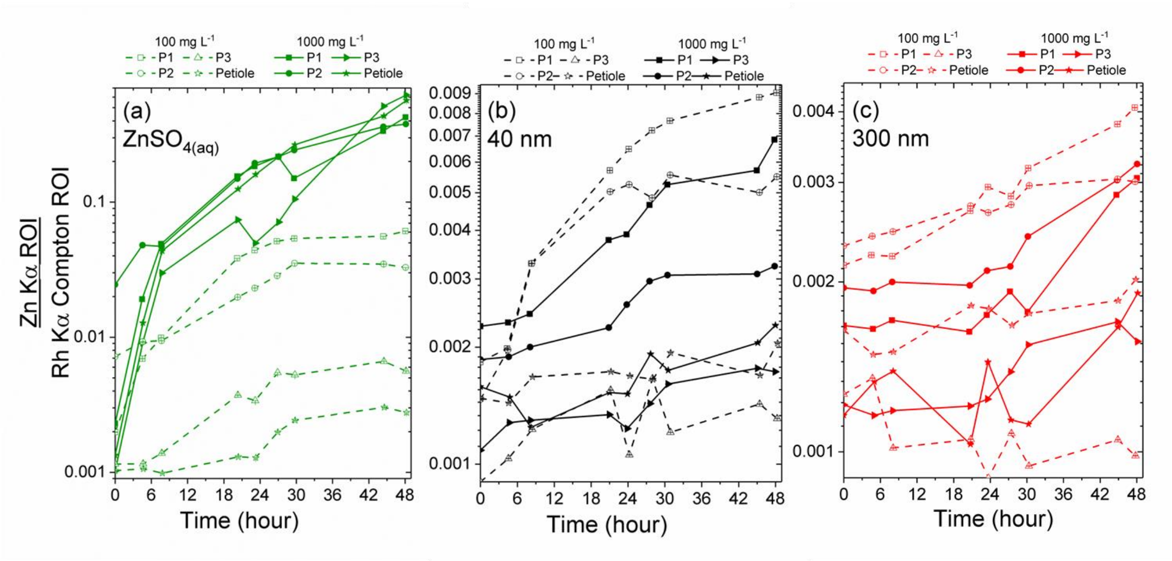

Figure S3. Second biologic replicate of the Zn uptake kinetics experiment. The Zn concentration was monitored in three points of the stem and one point in the petiole of *P. vulgaris* plants whose roots were immersed in 100 and 1000 mg Zn L^{-1} of $ZnSO_{4(aq)}$. Such as in Figure 2 of the main manuscript, the concentration of Zn decreased from root to shoot except for 1000 mg Zn L^{-1} $ZnSO_{4(aq)}$.

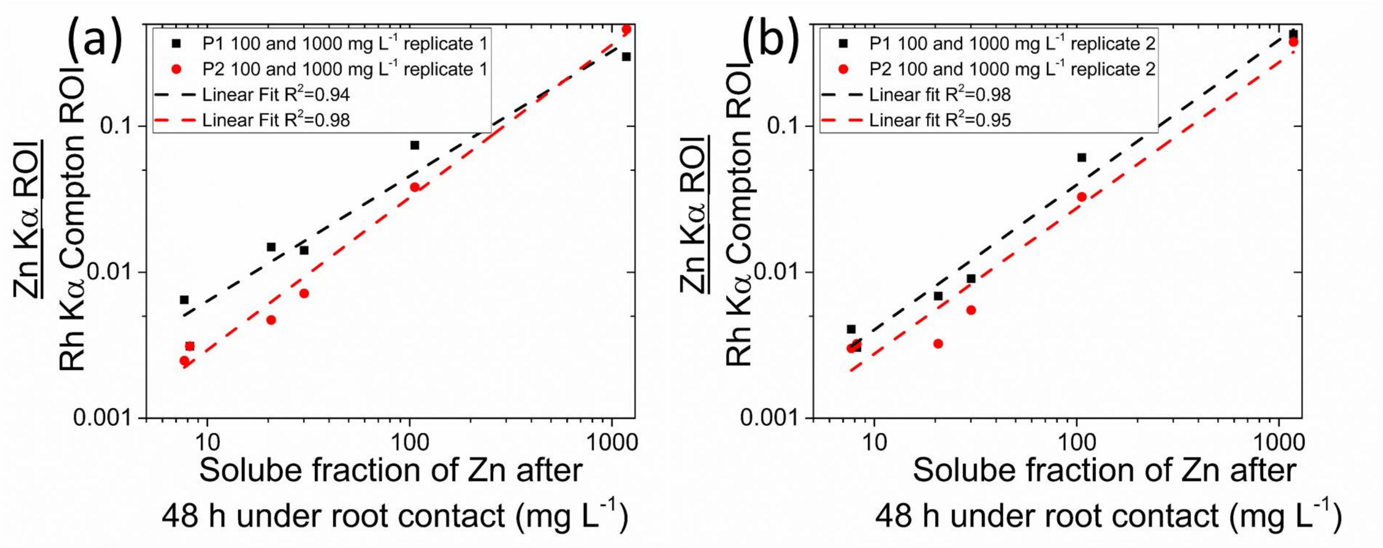

Figure S4. Zn content in P1 and P2 points of the stem as function of the concentration of Zn found in the dispersions and solution after 48 h. (a) first replicate data (same data as Figure 2 *vs* Table 1) and (b) second replicate (same data as Figure S3 *vs* Table 1). The Zn uptake presents a linear relationship with the concentration of soluble Zn.

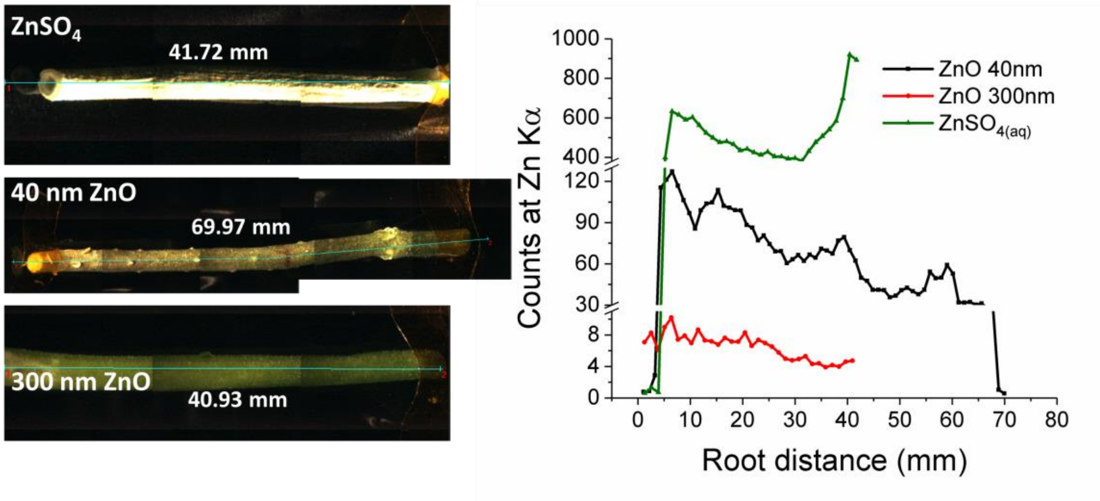

Figure S5. On the left panel are presented the stems of *P. vulgaris* treated with 1000 mg Zn L^{-1} for 48 hours. The green line indicates the X-ray beam path. The graph on the right shows total Zn Kα counts along the stem. The trend was the same observed in the kinetics shown in Figure 2 of the main manuscript. For 40 nm and 300 nm ZnO the Zn concentration decreases from root to shoot, this was more evident for the 40 nm ZnO treatment. $ZnSO_{4(aq)}$ treatment presented anomalous behaviour, curiously the concentration decreased and then suddenly increased form root to shoot.

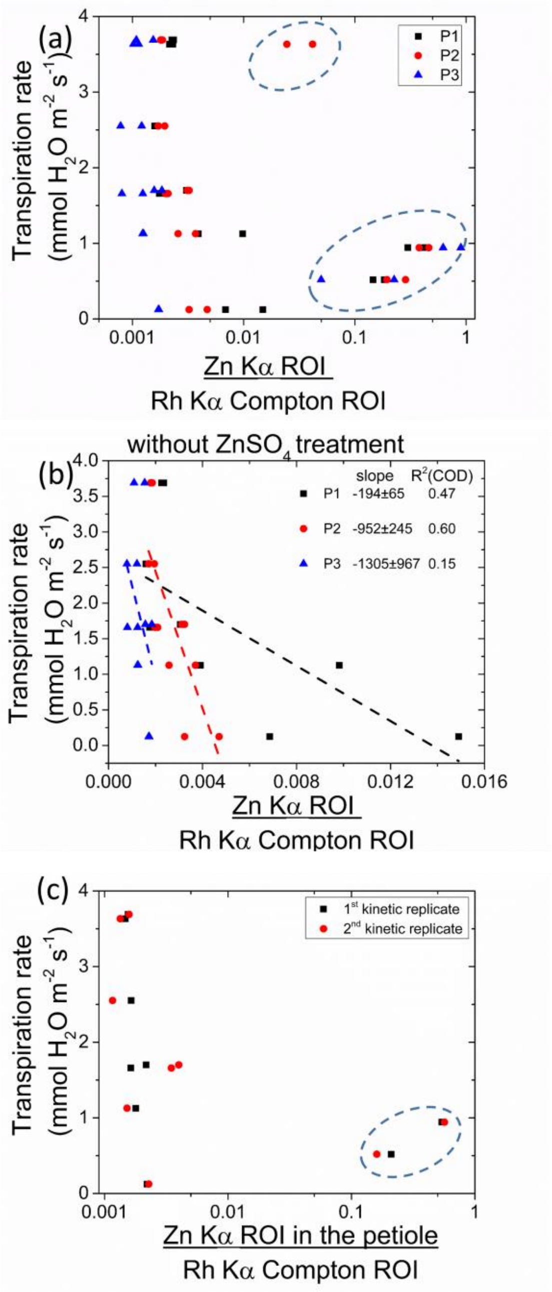

Figure S6. Transpiration rate determined by IRGA as function of the Zn content. (a) transpiration rate as function of Zn content in the points P1, P2 and P3 of the stem; (b) same data as the one shown in (a) excluding the $ZnSO_4$ treatment. The data for (a) and (b) correspond to the two replicates, however they are not specified in the Figures. (c) transpiration rate as function of the Zn content in the petiole. The points represent data recorded at 0, 24 and 48 h of root exposure to 1000 mg L^{-1} of $ZnSO_4$, 40 and 300 nm ZnO. The transpiration decreases as the Zn content increases. The two pairs of points highlighted by the circle correspond to the $ZnSO_4$ at 24 and 48 h of root exposure. The circles indicate the $ZnSO_4$ treatments, whose high concentrations made difficult access the effects of ZnO nanoparticles on the transpiration.

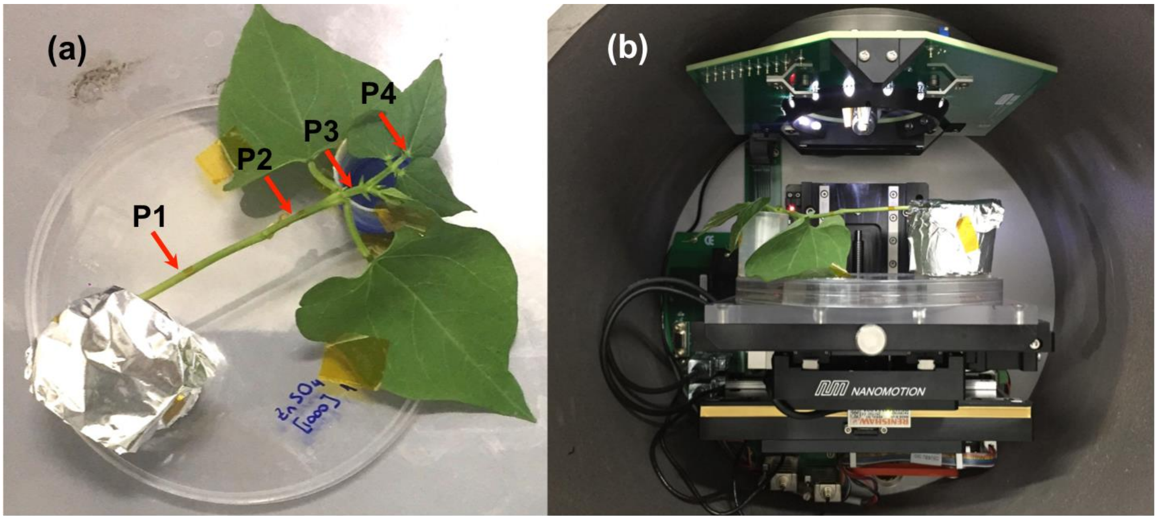

Figure S7. (a) Sample holder used in the *in vivo* Zn uptake with the 4 points analysed. (b) Setup used to monitor *in vivo* Zn absorption using a benchtop X-ray fluorescence equipment, where roots were always in contact with the treatments. The flask containing the treatments was covered with layers of aluminium foil to avoid any possible Zn XRF signal coming from the aqueous treatment excited by the scattered incoming beam.

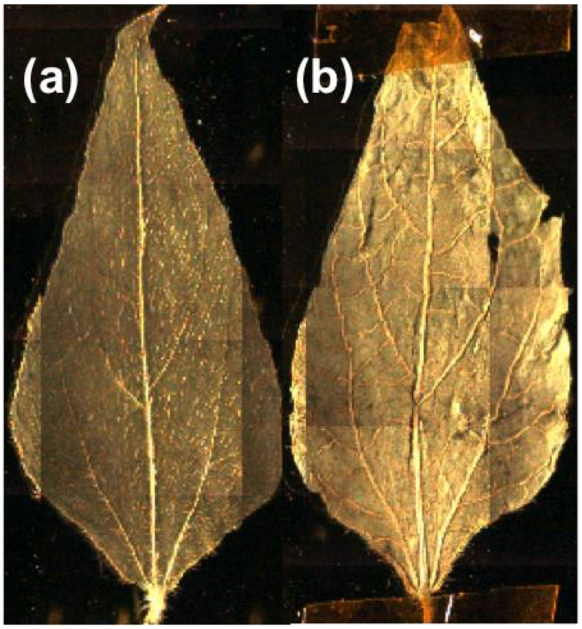

Figure S8. Mapped leaves treated with (a) 100 and (b) 1000 mg L^{-1} of $ZnSO_{4(aq)}$.

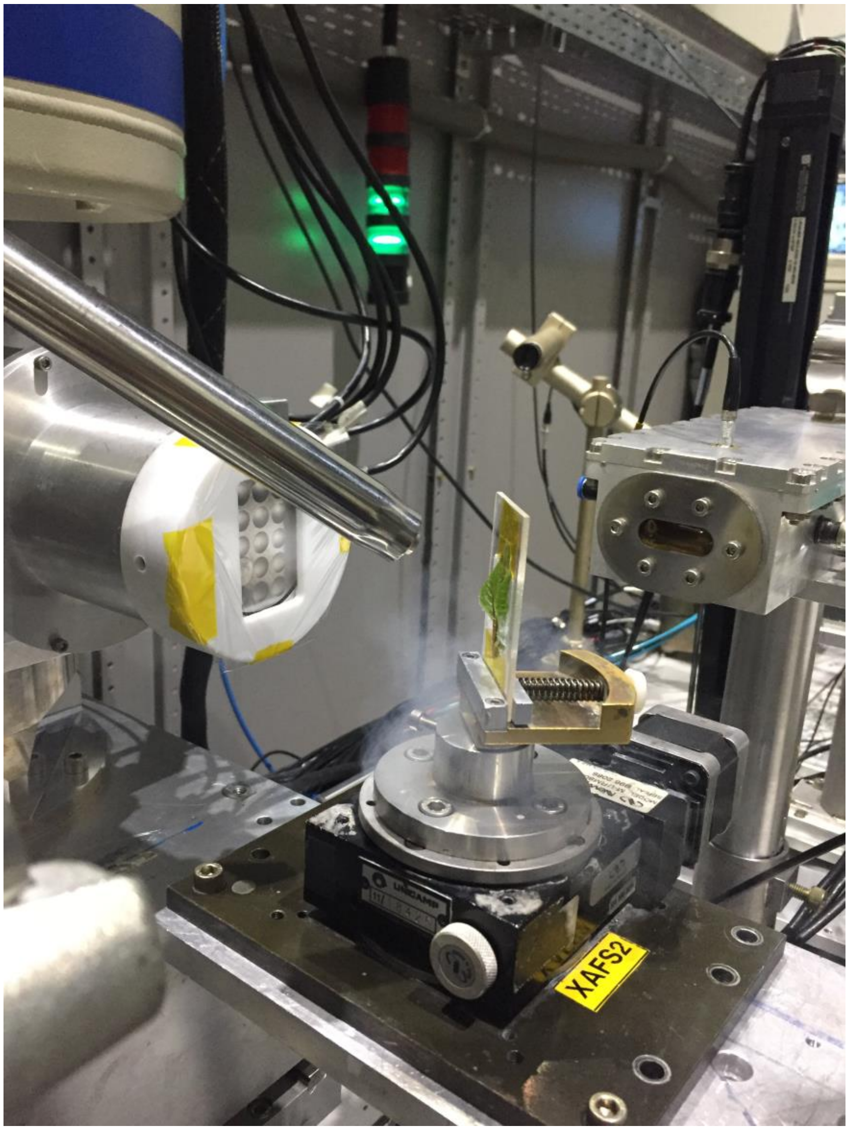

Figure S9. Sample holder with the *P. vulgaris* leaf assembled at XAFS2 beamline to record in fluorescence mode.

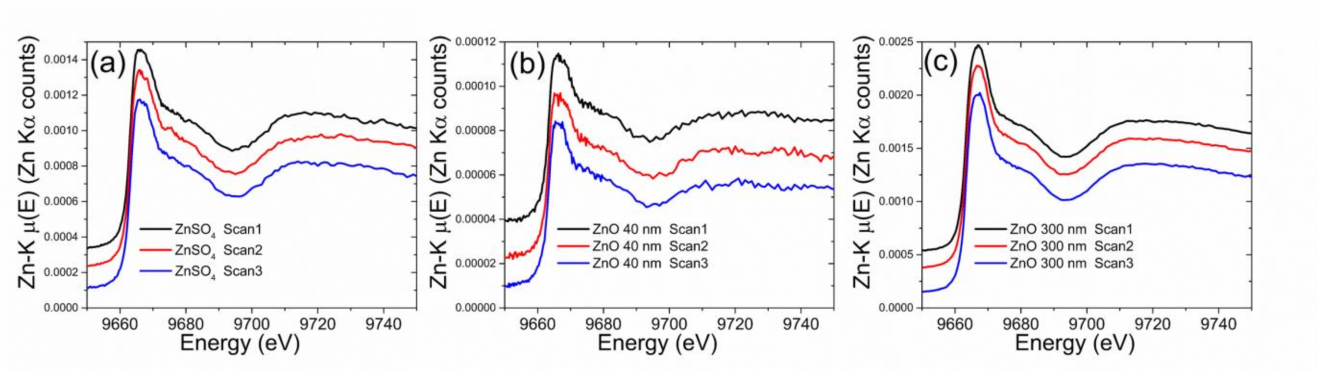

Figure S10. Non-normalized individual XANES scans recorded at the leaves of plants treated with 10 mg Zn L^{-1} for 7 days from (a) $ZnSO_4$, (b) ZnO 40 nm and (c) ZnO 300 nm. The absence of visual scorching and the stability/constancy of spectral features do not suggest radiation damage during the measurements.

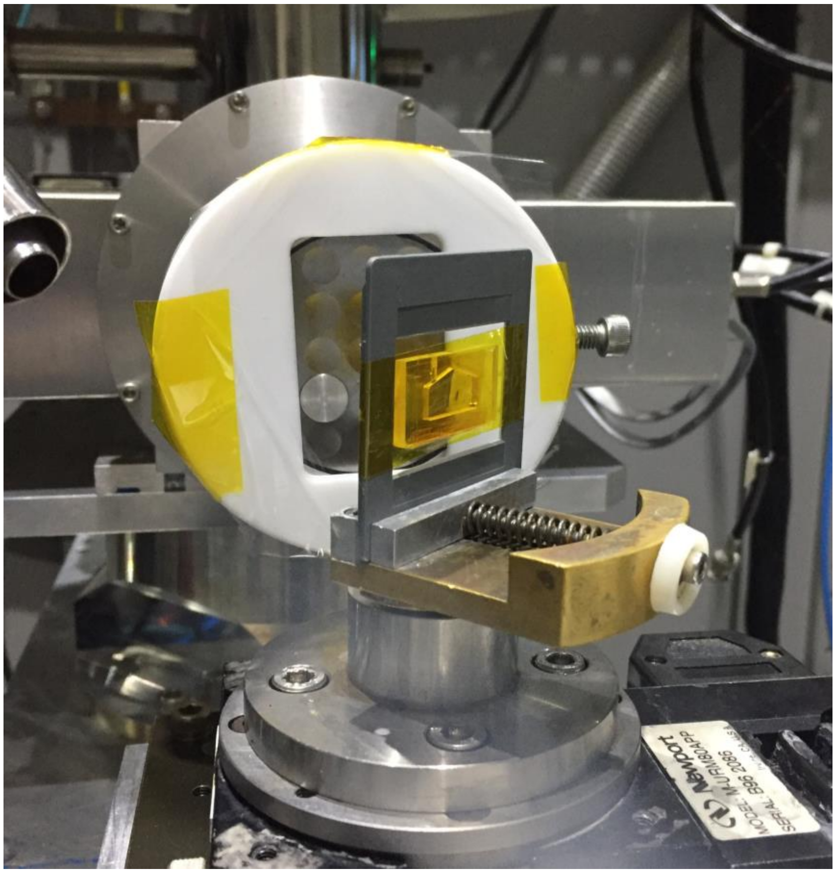

Figure S11. Sample holder with the aliquot from ZnO NPs dispersion to record XAS spectra in fluorescence mode.

Table S1. Physical chemical characterization of the supernatant dispersion.

	Prior to experiment (1000 mg L^{-1})		Supernatant suspension after 48h sedimentation (1000 mg L^{-1})			
			48h no root contact		48h under root contact (sonicated)	
	**40 nm**	**300 nm**	**40 nm**	**300 nm**	**40 nm**	**300 nm**
**$D_{(100)}$ (nm)**	29.9	15.8	#	15.2	#	#
**$D_{(002)}$ (nm)**	63.4	19.1	#	15.6	#	#
**$D_{(101)}$ (nm)**	40.5	20.7	#	19.9	#	#
**Average hydrodynamic diameter (nm)**	1060[B] (±40)	350[C] (± 20)	#	238[B] (±9)	1080[A] (± 70)	407[C] (±2)
**Zeta potential (mV)**	-8.8[B] (±1.6)	-26.6[C] (± 0.6)	-28.3[B] (± 1.5)	-28.3[B] (± 1.5)	-6.6[A] (±0.4)	-22[C] (±8)
**Suspended Zn concentration (mg Zn L^{-1})**	920 (± 50)	1360 (± 30)	34 (±11)	670	40 (± 20)	890 (±150)
**pH**	6.86 (± 0.08)	9.9 (± 0.3)	6.62 (± 0.08)	9.3 (± 0.2)	6.75 (± 0.13)	8.8 (± 0.3)

[A] Without dilution, [B] 10-fold dilution, [C] 100-fold dilution

No sufficient material for characterization

Table S2. Zn uptake velocity by *P. vulgaris* and Person's R from adjusted slopes as function of nanoparticle size, concentration and analysed part of the plants. Treatments consisted of 4 different points on the plants, 100 and 1000 mg L^{-1} of Zn from ZnO nanoparticles and aqueous $ZnSO_4$.

Treatments		100 mg L^{-1} Slope (counts min^{-1})	R^2	1000 mg L^{-1} Slope (counts min^{-1})	R^2
**Stem 1**	**40 nm**	2,83E-06	0,9833	1,55E-06	0,98062
	**300 nm**	6,53E-07	0,9783	3,93E-07	0,78852
	**$ZnSO_{4(aq)}$**	2,41E-05	0,9723	1,19E-04	0,97912
**Stem 2**	**40 nm**	1,53E-06	0,905	5,28E-07	0,9541
	**300 nm**	2,59E-07	0,9706	4,08E-07	0,87257
	**$ZnSO_{4(aq)}$**	1,32E-05	0,9592	1,23E-04	0,98745
**Stem 3**	**40 nm**	1,40E-07	0,6798	2,12E-07	0,91167
	**300 nm**	-2,15E-08	-0,194	1,73E-07	0,88069
	**$ZnSO_{4(aq)}$**	2,06E-06	0,9576	5,03E-05	0,82798
**Petiole**	**40 nm**	1,44E-07	0,7762	2,84E-07	0,84036
	**300 nm**	1,43E-07	0,8475	6,82E-08	0,2569
	**$ZnSO_{4(aq)}$**	5,74E-07	0,8581	1,32E-04	0,95737

Table S3. Average Zn concentration in the shoot and root of *P. vulgaris* plants. The long-term exposure using lower concentration, i.e. 10 mg L^{-1} treatment was able to increase the Zn concentration, while none visual symptoms of intoxication were observed. A control treatment was analyzed and it presented 54, and 62 mg Zn kg^{-1} in shoot and root, respectively. The concentration measured for roots include both Zn inside and adsorbed outside of the roots.

Treatment	Shoot			Root	
	100 mg L^{-1} for 2 days	1000 mg L^{-1} for 2 days	10 mg L^{-1} for 7 days	100 mg L^{-1} for 2 days	1000 mg L^{-1} for 2 days
**$ZnSO_4$**	363	19,473	1,213	4,192	11,429
**ZnO 40 nm**	94	111	759	980	29,110
**ZnO 300 nm**	100	105	1,283	6,080	85,729

Table S4. Linear combination fittings for the XANES spectra recorded at the leaves of common beans (*Phaseolus vulgaris*) treated with $ZnSO_4$ and differently sized ZnO NP. For the leaves treated with 300 nm ZnO we observed a slight decrease of the white line intensity for the third spectrum. Therefore, we present the retrieved percent composition found by two distinct strategies: i) merging three scans or ii) using only the first scan. The result shows that regardless the strategy the values fall within the error bar range.

Leaf sample	Percent composition			R-factor[a] x10^{-3}
	$Zn_3(PO_4)_2$	Zn-histidine	Zn-malate	
**$ZnSO_4$ 3 merged scans**	62±3	38±3	-	1.8
**$ZnSO_4$ first scan**	69±3	31±4		3.1
**ZnO 40 nm 3 merged scans**	82±4	18±4	-	3.5
**ZnO 40 nm first scan**	87±3	13±4		2.0
**ZnO 300 nm 3 merged scans**	73±1	-	27±2	0.9
**ZnO 300 nm first scan**	73±1		27±1	0.9
**ZnO 300 nm 3 merged scans**	59±2	11±3	30±1	0.7

[a] The linear combination disagreement expressed as “R-factor” is the squared sum over all mismatch within the selected region between -20 and 50 eV relatively to edge. It is given by the formula below. For details refer to Calvin, S. *XAFS for Everyone*; Taylor & Francis, 2013.

.

$$R - factor = \frac{\sum(data_i - fit_i)^2}{\sum(data_i)^2}$$

Table S5. List of primer sequences for detection of mRNA levels of transporter genes by using the quantitative RT-PCR.

Gene	TAIR10 ID (*Arabidopsis*)	Phytozome ID* (*P. vulgaris*)	Primer	5'–3'sequence
**HMA2**	AT4G30110	Phvul.003G142700	PvHMA2-qRT-PCR-F<br>PvHMA2-qRT-PCR-R	GGTGGCACAGGTGTTTTGATC<br>ACCCAGCACATCAAAGTAGC
**NRAMP3**	AT2G23150	Phvul.003G238600	PvNRAMP3-qRT-PCR-F<br>PvNRAMP3-qRT-PCR-R	TCAACCTCTCTTCTCACTGCAC<br>GGATTCGTAGGCTGTTTCTTGC
**NRAMP4**	AT5G67330	Phvul.002G014300	PvNRAMP4-qRT-PCR-F<br>PvNRAMP4-qRT-PCR-R	TGGGCAGTTTCAGAATTGGC<br>ACTGCTCCATTCACTTCAGAGG
**MTP1**	AT2G46800	Phvul.010G119900	PvMTP1-qRT-PCR-F<br>PvMTP1-qRT-PCR-R	TTGAGCGGGGATTGCAATTG<br>TGCGTTTGCTGAGAGCTTTG
**MTP8**	AT3G58060	Phvul.008G244200	PvMTP8-qRT-PCR-F<br>PvMTP8-qRT-PCR-R	CTGAAGTTCTGCAGAAGCTGAC<br>AATCCTCCGGCAGTTCAATG
**ZIF1**	AT5G13750	Phvul.002G108300	PvZIF1-qRT-PCR-F<br>PvZIF1-qRT-PCR-R	CGGTGCTCAAACGCAATATG<br>AGCTCCCCACAACAAAGATG
**IRT3**	AT1G60960	Phvul.009G077700	PvIRT3-F<br>PvIRT3-R	AGAATAACACCATCCCCAAAATTA<br>AGTCACTATGGGAATGTCACAGAA

*Homologs of *Arabidopsis* gene in *P. vulgaris* genome

Uncertainty of XRF measurements

The error of each XRF measurement, equivalent to one standard deviation (σ) was determined using the equation below:

$$\sigma = \frac{\sqrt{\frac{Zn\ K\alpha\ net\ count\ rate\ \ (cps)}{t\ (s)}}}{Rh\ K\alpha\ Compton\ counts}$$

cps stands for counts per second and s means seconds.

It means that the uncertainty decreases as function of time. During the optimization of the instrumental parameters, we concluded that no meaningful gain on the analytical quality would be obtained increasing the measurement time above 120 s.
